# The Effects of 30 Minutes of Artificial Gravity on Cognitive and Sensorimotor Performance in a Spaceflight Analog Environment

**DOI:** 10.3389/fncir.2022.784280

**Published:** 2022-03-02

**Authors:** Grant D. Tays, Heather R. McGregor, Jessica K. Lee, Nichole Beltran, Igor S. Kofman, Yiri Eleana De Dios, Edwin Mulder, Jacob J. Bloomberg, Ajitkumar P. Mulavara, Scott J. Wood, Rachael D. Seidler

**Affiliations:** ^1^Department of Applied Physiology and Kinesiology, University of Florida, Gainesville, FL, United States; ^2^German Aerospace Center (DLR), Cologne, Germany; ^3^KBR, Houston, TX, United States; ^4^NASA Johnson Space Center, Houston, TX, United States; ^5^Norman Fixel Institute for Neurological Diseases, University of Florida, Gainesville, FL, United States

**Keywords:** sensorimotor, cognition, artificial gravity, head-down tilt bed rest, spaceflight

## Abstract

The altered vestibular signaling and somatosensory unloading of microgravity result in sensory reweighting and adaptation to conflicting sensory inputs. Aftereffects of these adaptive changes are evident postflight as impairments in behaviors such as balance and gait. Microgravity also induces fluid shifts toward the head and an upward shift of the brain within the skull; these changes are well-replicated in strict head-down tilt bed rest (HDBR), a spaceflight analog environment. Artificial gravity (AG) is a potential countermeasure to mitigate these effects of microgravity. A previous study demonstrated that intermittent (six, 5-mins bouts per day) daily AG sessions were more efficacious at counteracting orthostatic intolerance in a 5 day HDBR study than continuous daily AG. Here we examined whether intermittent daily AG was also more effective than continuous dosing for mitigating brain and behavioral changes in response to 60 days of HDBR. Participants (*n* = 24) were split evenly between three groups. The first received 30 mins of continuous AG daily (cAG). The second received 30 mins of intermittent AG daily (6 bouts of 5 mins; iAG). The third received no AG (Ctrl). We collected a broad range of sensorimotor, cognitive, and brain structural and functional assessments before, during, and after the 60 days of HDBR. We observed no significant differences between the three groups in terms of HDBR-associated changes in cognition, balance, and functional mobility. Interestingly, the intermittent AG group reported less severe motion sickness symptoms than the continuous group during centrifugation; iAG motion sickness levels were not elevated above those of controls who did not undergo AG. They also had a shorter duration of post-AG illusory motion than cAG. Moreover, the two AG groups performed the paced auditory serial addition test weekly while undergoing AG; their performance was more accurate than that of controls, who performed the test while in HDBR. Although AG did not counteract HDBR-induced gait and balance declines, iAG did not cause motion sickness and was associated with better self-motion perception during AG ramp-down. Additionally, both AG groups had superior cognitive performance while undergoing AG relative to controls; this may reflect attention or motivation differences between the groups.

## Introduction

Human spaceflight has been shown to result in numerous transient effects on human performance when crewmembers return to Earth. Sensorimotor declines have been demonstrated in locomotion (McDonald et al., 1996; Bloomberg et al., 1997; Layne et al., 1998), balance (Paloski et al., 1992, 1994; Reschke et al., 1994a,b, 1998; Black et al., 1995, 1999), jump landing (Newman et al., 1997), fine motor control (Lackner and DiZio, 1996) and obstacle navigation (Mulavara et al., 2010; Bloomberg et al., 2015) following spaceflight. There are also changes in sensory perception (Kornilova, 1997; Clément et al., 2013; Lowrey et al., 2014), and relative weighting of sensory inputs (Lowrey et al., 2014). Astronauts have also reported feelings of “space fog” inflight (Welch et al., 2009) including mental slowing, troubles concentrating and impaired cognitive performance (Kanas and Manzey, 2008; Clément et al., 2020). Manzey et al. (1995) and Manzey and Lorenz (1998) reported declines in astronauts’ abilities to perform cognitive and motor dual tasking early in spaceflight that stabilized over the duration of the mission, while Garrett-Bakelman et al. (2019) reported increased risk taking, decreased accuracy in a visual object learning task and decreased abstract matching in the NASA Twins Study.

Short duration shuttle missions lasted roughly 2 weeks, and with the completion of the International Space Station (ISS), mission duration increased to ∼6 month missions (with some up to 1 year). As NASA sets their goals for human travel to the moon and beyond to Mars, flight duration will further increase to an expected ∼30 months (Clément et al., 2020). With the increased duration from shuttle to ISS missions, sensorimotor deficits post-flight increased in their duration as well (Miller et al., 2018). Thus, there is a need for countermeasures to mitigate the negative effects of microgravity on human performance and physiology. Astronauts already engage in physical activity aboard the ISS, somewhat mitigating postflight sensorimotor declines (Wood et al., 2011). Exercise alone does not completely prevent these changes, however, requiring further development of countermeasures (Mulavara et al., 2018; Ploutz-Snyder et al., 2018). There are also other physiological and functional changes with microgravity, including brain position shifts, decreases in bone and muscle mass, and orthostatic intolerance, among others (Buckey et al., 1996; Adams et al., 2003; Roberts et al., 2017; Stavnichuk et al., 2020). There are numerous countermeasures under investigation for these changes individually; by contrast, artificial gravity (AG) could provide a single, integrated countermeasure by “replacing” Earth’s gravitational effects on the body (Clément et al., 2015).

In head-down tilt bed rest (HDBR) studies, participants lie at 6° with their head below their feet; this simulates the head-ward fluid shift and the axial body unloading of the microgravity environment. HDBR models several of the physiological effects of microgravity, such as arterial pressure changes, unloading of muscles and fluid changes (Reschke et al., 2009; Mulder et al., 2014; Koppelmans et al., 2017; Hargens and Vico, 2016; Miller et al., 2018; Mulavara et al., 2018). Sensorimotor performance declines are also exhibited following HDBR; multiple studies have shown that post-HDBR postural stability and functional mobility declines are similar to those exhibited post-flight (Reschke et al., 2009; Mulder et al., 2014; Koppelmans et al., 2017; Miller et al., 2018; Mulavara et al., 2018; Lee et al., 2019). Due to this, HDBR serves as an effective analog environment in which to investigate spaceflight countermeasure efficacy.

Artificial gravity is implemented on Earth as short-radius centrifugation, resulting in centripetal acceleration along the long axis of the body. AG has been shown to be generally well tolerated in humans, with one study showing over 97% of total sessions were completed without incident during 21 days of 6° HDBR where participants received 60 continuous minutes of daily centrifugation (Arya et al., 2007). Additionally, higher magnitudes of centrifugation (3+ G along a supine subject’s X-axis) have been shown to result in similar visual-vestibular changes in otolith function tests as astronauts following a Spacelab mission (Bles et al., 1997). More recently, it has been shown that intermittent (six bouts of 5 mins) AG reduces orthostatic intolerance following 5 days HDBR to a greater extent than 30 mins of continuous AG (Linnarsson et al., 2015) while also being more highly tolerated by participants (Clément et al., 2015). In the same AG campaign as our current investigation (Artificial Gravity Bed Rest – European Space Agency or “AGBRESA”), it was concluded that participants sufficiently tolerated both AG conditions, continuous and intermittent, but that the intermittent group tolerated it marginally better as evidenced by heart rate and blood pressure recorded while in the centrifuge, motion sickness scores and rating of perceived exertion immediately following centrifugation (Frett et al., 2020). Participants underwent 960 centrifuge runs overall, with only 10 runs being terminated early (eight continuous); only one termination was a result of severe motion sickness (Frett et al., 2020). Overall, AG participants reported similar levels of perceived exertion, sleepiness, affect scores on the Positive and Negative Affect Schedule (PANAS) test, and cardiovascular reactions (Frett et al., 2020).

Moore et al. (2010) aimed to investigate the effects of 21 days of 6° HDBR combined with 60 continuous minutes of daily AG on spatial orientation measured in the upright position. Compared to controls that did not receive AG, AG participants showed no direct effects of AG or HDBR, but demonstrated more errors on a spatial orientation test for 48 h after exiting HDBR (Moore et al., 2010). In a separate study, participants underwent 21 days of HDBR at 6°, where they either received 1 h of continuous AG via centrifugation per day or received no AG (Seaton et al., 2007). The participants that underwent AG had more off-nominal scores (75% of the total) on the WinSCAT cognitive assessment than controls did (25% of the total), as assessed immediately following AG. However, with a small sample size it is uncertain whether performance declines were due to centrifugation or reduced motivation and heightened distraction in the AG participants (Seaton et al., 2007). AG has also been shown to have no negative effect on sensorimotor performance (Lackner and DiZio, 1998; DiZio and Lackner, 2002), however, it did not appear to increase performance either when assessed through application of Coriolis forces generated from room rotation speeds up to 20 rpm. Thus, studies that have applied 1 h of AG have shown detrimental, acute effects on cognitive function.

Here, we administered a similar battery of cognitive and sensorimotor assessments as we have previously applied in our HDBR and spaceflight studies (Koppelmans et al., 2017; Cassady et al., 2016; Yuan et al., 2016, 2017, 2018a,2018b; Hupfeld et al., 2019, 2020; McGregor et al., 2020, 2021; Salazar et al., 2020, 2021; Banker et al., 2021). Our primary aim in the current study was to examine whether centrifugal artificial gravity applied along the long axis of the body at approximately 1G at the center of mass modulates the effects of HDBR on sensorimotor and cognitive performance. We hypothesized that (1) 30 mins of daily artificial gravity would at least partly mitigate the cognitive and sensorimotor performance declines occurring with 60 days of HDBR relative to HDBR controls who do not undergo AG; and (2) participants that receive AG intermittently (six, 5 min bouts per day) would perform at a similar level as those that receive AG continuously (one, 30 min bout per day) while tolerating AG better.

## Materials and Methods

### Participants

Twenty four (8 F, 33.3 ± 9.17 years, 174.6 ± 8.6 cm, 74.2 ± 10.0 kg) participants volunteered for this study and were assigned to one of three groups. Two groups received centrifugal artificial gravity applied either (1) continuously in one 30-min bout daily (cAG); or (2) intermittently in six bouts of 5 min with 3 mins between each bout, daily (iAG). The third group served as a control group (Ctrl) that received no artificial gravity. All subjects were familiarized with AG twice (BDC-11 and BDC-4) during the baseline phase, prior to being separated into groups. Participants were screened for AG tolerance to ensure they would be able to complete centrifugation. They were also selected to be as close as possible in age, sex and education level to astronauts, yet it was not an exclusion criteria. Three participants exited the study early and were subsequently replaced; their partial data sets are not considered here. All participants provided their written informed consent. The University of Florida and NASA Institutional Review Boards as well as the local ethical commission of the regional medical association (Ärztekammer Nordrhein) approved all study procedures. Informed consent was obtained from all participants. They underwent 60 days of 6° strict HDBR and performed a range of sensorimotor and cognitive tasks both in and out of the centrifuge at multiple time points prior to, during, and following the 60 day protocol. The measures implemented in this study overlap with those in our ongoing NASA supported flight and prior bed rest studies (Koppelmans et al., 2013; Cassady et al., 2016; Yuan et al., 2016, 2017, 2018a,2018b; Hupfeld et al., 2019, 2020; Lee et al., 2019; McGregor et al., 2020, 2021; Salazar et al., 2020, 2021; Banker et al., 2021; Mahadevan et al., 2021).

### Head-Down Tilt Bedrest

Participants were maintained in a strict 6° head-down tilt for 24 h per day as per the International Guidelines for Standardization of Bed Rest Studies in the Spaceflight Context.^1^ They were allowed to maintain this either on their back or side, but performed all activities and hygiene maintenance in these positions. Transportation to and from testing facilities within the building was conducted on a specially designed gurney that maintained the HDBR position. For 14 days prior to bedrest and 14 days following, participants were kept under observation at the:envihab facility to restrict free movement and reduce confounding behavior. Participants were kept on a controlled diet that was strictly enforced and had biometrics frequently monitored.

### Artificial Gravity

Artificial gravity was applied through the Deutsches Zentrum für Luft- und Raumfahrt German Aerospace Center’s (DLR) short-arm human centrifuge with a radius of 3.8 m (see Frett et al., 2020). Rotational speed of the centrifuge was set to maintain an acceleration of 1g at the center of mass and approximately 2g at the feet along the long axis (Gz) of the body. Speeds ranged from 29.1 rotations per minute for the tallest subjects, to 32.2 rotations per minute for the shortest subjects. Participants were instructed to remain in the supine position with their head toward the center of the centrifuge and avoid head movement while in the centrifuge. Rotation direction was alternated daily within group so that half of the participants in a group were spun clockwise and the other spun counter-clockwise per day. There was a medical team on site supervising.

### Behavioral Assessments

#### Sensorimotor Assessments

##### Functional Mobility Test

To assess overall mobility, participants performed the Functional Mobility Test (FMT; Mulavara et al., 2010; Koppelmans et al., 2013). The FMT is a short obstacle course, measuring 6 × 4 m that the participant must move around, under and over foam obstacles and both firm and soft surfaces from a seated position. This was designed by NASA to measure astronauts’ mobility in order to gauge their ability to rapidly egress in emergency situations. We examined the total completion time for the course on the first of 10 trials, to minimize the influence of motor learning in our analysis.

##### Computerized Dynamic Posturography

To assess dynamic postural control, we utilized computerized dynamic posturography (Equitest, NeuroCom International, Clackamas, OR, United States; Reschke et al., 2009). This assessment includes several sensory organization tests (SOT); equilibrium scores are calculated from the peak-to-peak excursion of the center of mass (estimated at 55% of total height, Wood et al., 2012). This is administered through three, 20-s trials (Nashner, 1972; Paloski et al., 1992). We administered the SOT-5 with eyes closed on a sway-referenced platform that forces more reliance on vestibular afferent inputs and the SOT-5M during which the participant makes ±20° head pitch movements at 0.33 Hz paced by auditory tones (Wood et al., 2015). The median scores of the three trials for SOT-5 and SOT-5M were used for statistical analyses.

##### Purdue Pegboard Test

To assess bimanual coordination we measured completion time on the bimanual condition of the Purdue Pegboard test (Tiffin and Asher, 1948). The Purdue Pegboard assesses manual dexterity through measuring the time it takes to place 15 small, metal pegs into fitted holes with the two hands. Total completion time was recorded and used for statistical analysis.

#### Cognitive Assessments

##### Spatial Working Memory

We also administered multiple tests of cognitive function, including measures of processing speed, mental rotation, and spatial working memory. We used three tasks to probe spatial working memory performance; (1) a spatial working memory task (SWM; Anguera et al., 2010), (2) Thurstone’s 2D card rotation test (Ekstrom and Harman, 1976) and (3) a three dimensional cube task (Shepard and Metzler, 1988). While performing the SWM task, participants had to mentally connect three dots on a screen that formed a triangle. The dots would disappear for a short retention phase (3 s) then three new dots would appear and the participants would be required to identify if it was the same tringle rotated, or a different triangle (Anguera et al., 2010; Salazar et al., 2020). They also performed a control task, where instead of seeing a second pair of dots, they would see a single dot with a very short retention period (200 ms) and have to identify if that single dot was one of the previously presented three dots. They performed 30 trials, and response time and accuracy of responses were measured and analyzed. While performing the 2D card rotation task, participants were presented with a two dimensional drawing of an abstract shape. They were then given eight new drawings and asked to identify if the new drawings were a rotated or mirrored version of the original drawing (Ekstrom and Harman, 1976; Salazar et al., 2020). To assess their performance, we recorded completion time, amount completed (if the test was not completed in 3 mins), and accuracy. For the 3D cube rotation task, participants were presented with a three-dimensional image of a cube assembly, created from stacked smaller cubes, for 3 s. Followed by a 2 s retention phase, two new cube assemblies would appear and the participants must identify as quickly as possible which of the two figures was the original, yet rotated cube assembly (Shepard and Metzler, 1988; Salazar et al., 2020). Reaction time and accuracy were measured and analyzed.

##### Digit Symbol Substitution Task

To assess processing speed we measured completion time and accuracy on the digit symbol substitution task (DSST; Weschler, 1986). During the DSST, participants are presented with a sheet of paper that requires them to match numbers with symbols according to a key at the top and to “decode” a variety of symbols on paper.

##### Rod and Frame Test

We used the Rod and Frame Test (RFT) to assess visual dependence for perception (Witkin and Asch, 1948). During the RFT participants must align a rod to their perception of Earth’s vertical. The rod is viewed within a frame, both of which may be tilted relative to vertical. The participant views the screen by looking in a “tunnel,” thus removing any room visual cues. We used the frame effect and response consistency (a measure of variability) to test for any changes in visual dependence.

##### Cognitive-Motor Dual Tasking

We also assessed performance on cognitive-motor dual tasking. Participants were instructed to monitor a visual display. An “X” would appear in a small box either to the left or right of the center of the screen, indicating the respective response buttons that should be pressed for a given trial (right side, right button, etc.). During the cognitive task, participants monitored a separate, visual display box that appeared on the same screen, immediately above the response button boxes which rapidly changed colors; they were instructed to count the number of times the box turned blue. This occurred rarely making the task comparable to an oddball task. Both of these tasks were performed individually (in a single task condition), and combined (in a dual task condition) prior to performing the dual task condition. For analysis, performance declines from single task (ST) to dual task (DT) were calculated as dual task cost (DTC; (DT − ST)/ST × 100). DTC can be used as a marker of central processing capacity (Tombu and Jolicoeur, 2003), as a higher DTC would suggest interference and higher processing loads. We have previously used DTC to analyze changes in HDBR analogs (Yuan et al., 2016, 2017).

#### Peri-Centrifugation Assessments

##### Paced Auditory Serial Addition Test

To assess performance during centrifugation we administered a selection of tasks during and immediately following centrifugation on a weekly basis; controls performed the same tasks in bed using the same, weekly timeframe. cAG and iAG performed the Paced Auditory Serial Addition Test (PASAT) (Gronwall, 1977) during the last 5 mins of their centrifugation session. During this, the participants listened to a recording that presented pseudo-random, numerical stimuli every 3 s. They were instructed to continuously sum the previous two numbers and verbally respond with an answer. This was performed during centrifugation, and reaction time and accuracy were measured for statistical analysis.

##### Motion Sickness and Illusory Motion

To assess performance in artificial gravity, we also measured (1) post-AG illusory motion and (2) motion sickness responses. Post-AG illusory motion was recorded immediately following centrifugation with the participants in the dark and with their eyes closed. As participants were coming to a stop, they were instructed to press a button when they perceived that they were no longer moving, or that they perceived the direction of their rotation had reversed. This was measured as the time difference between the actual stop of the centrifuge and the button press. Negative values indicate the participant has perceived that they have come to a stop before they actually have, while positive values indicate the time it takes for them to perceive they have come to a stop after they have physically stopped.

We used the Motion Sickness Assessment Questionnaire (MSAQ; Gianaros et al., 2001) to assess motion sickness immediately following centrifugation in those that received AG. Participants were asked a short list of questions and responded along a 1–9 (9 being the most severe) scale. Control subjects were assessed on the same day as AG, yet in their normal setting. The average value of these responses were utilized to assess motion sickness.

#### Timeline

**FIGURE 1:**
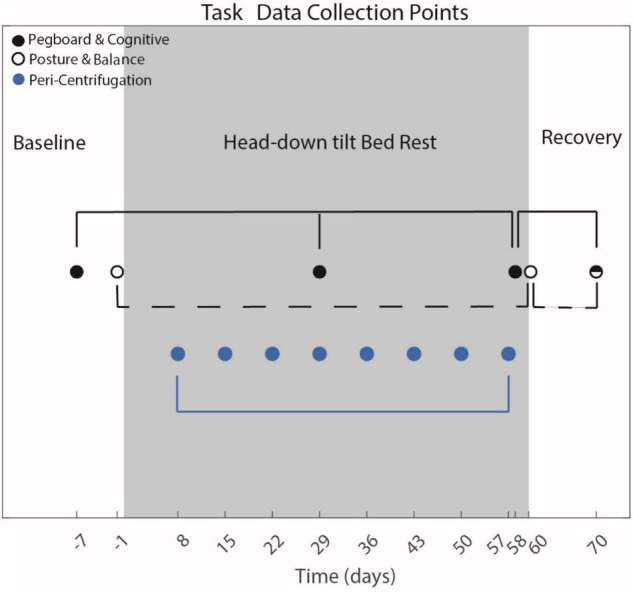
Testing Timeline. Entering **head-down tilt bed rest** (HDBR) occurred on day 0. Cognitive and sensorimotor measures were collected at various points before, during and following HDBR. Peri-centrifugation tasks only occurred during HDBR. Filled black dots represent data collection time points for the Purdue Pegboard and cognitive assessments. The open filled black circles represent the data collection time points of the posture and balance tasks. The half-filled dot indicates that all sensorimotor and cognitive assessments were collected at that time. The blue dots represent the peri-centrifugation task collections. The day relative to entering HDBR upon which the data were collected is plotted. Only data collection time points used for statistical analysis are included in this figure.

Sensorimotor and cognitive tasks were measured before, during and following HDBR + AG (Figure 1). The bimanual Purdue Pegboard test and all cognitive tasks were measured 7 days prior to entering HDBR, on day 29 and 58 of HDBR and 10 days post HDBR. The FMT and SOT were not administered during this same timeline as they require upright stance. Thus, they were assessed pre- and post-HDBR. The FMT was collected on BDC-7, and the day of exiting HDBR (R + 0). The SOT-5 was collected 1 day prior to entering HDBR (BDC-1) and on R + 0. Peri-centrifugation measures were administered on approximately days 8, 15, 22, 29, 36, 43, 50, and 57 of HDBR (i.e., weekly).

#### Statistical Analyses

We used the nlme package (Pinheiro et al., 2022) in R 3.6.1 (R Core Team, 2019) to fit linear mixed effects models with restricted maximum likelihood (REML) to test for changes over time. Within each model, subject was entered as the random intercept to allow for different starting points of each participant (as we did in previous work Koppelmans et al., 2017). We evaluated three models to examine group differences in: (1) the effect of the HDBR + AG environment, (2) recovery from the HDBR + AG environment and (3) the direct effects of centrifugation. In the first and second models the two artificial gravity groups were combined to increase statistical power. Only statistically significant effects from model 1 were included in model 2 to assess their recovery following HDBR + AG. In several cases the data were not normally distributed; we addressed this by log transforming the data prior to statistical analyses. We corrected for multiple comparisons within each model with the Benjamini-Hochberg false discovery rate correction (Benjamini and Hochberg, 1995); findings are presented in Tables 1–3.

**TABLE 1:** Effect of HDBR + AG environment.

HDBR + AG		HDBR		Group		HDBR × AG		Age		Sex	
Sensorimotor Task		β	*p*	β	*p*	β	*p*	β	*p*	β	*p*
Pegboard	Time (s)	0.076	0.283	–2.525	0.373	0.418	0.209	1.379	**0** . **005**	–0.051	0.591
FMT	Time (s)	0.139	**0.0002**	0.220	0.914	–0.029	0.448	0.173	0.074	–7.443	**0.0004**
SOT-5	EQ Score	–0.055	0.660	1.229	0.805	–0.078	0.284	–0.141	0.546	5.785	0.205
SOT-5M	EQ Score	–0.609	**0.0002**	–0.120	0.985	0.179	0.143	–0.112	0.687	2.497	0.634

**Cognitive Task**		β	***p***	β	***p***	β	***p***	β	***p***	β	***p***

DSST	Time (s)	0.013	0.958	10.614	0.517	–0.163	0.257	2.667	**0** . **005**	11.679	0.462
Card rotation	Time (s)	–0.039	0.826	–7.148	0.441	–0.084	0.408	0.460	0.318	–6.556	0.456
	Correct (%)	0.084	0.345	10.017	0.049	0.001	0.914	–0.674	**0.010**	4.891	0.297
	Compl. (%)	0.045	0.582	8.743	0.064	0.021	0.658	–0.603	**0.014**	4.310	0.325
RFT	Variability	0.019	0.608	–1.751	0.067	–0.014	0.524	–0.059	0.126	–0.020	0.978
	Frame Effect	–0.038	0.273	2.758	0.191	0.022	0.272	0.151	0.157	0.803	0.687
Cube Rotation	Time (s)	0.002	0.768	0.728	0.059	–0.001	0.753	0.028	0.135	0.213	0.543
	Correct (#)	0.204	0.578	1.079	0.368	0.001	0.948	–0.043	0.429	1.089	0.302
DTC	Tap	–0.055	0.246	–1.225	0.409	0.032	0.244	–0.162	0.023	2.262	0.088
	RT	0.029	0.695	0.031	0.990	–0.016	0.722	0.076	0.452	–1.767	0.362
	Count	0.934	0.551	0.152	0.887	0.024	0.868	0.171	0.533	–0.379	0.942
SWM	Rotation Correct (#)	0.011	0.768	0.760	0.505	–0.004	0.847	–0.137	**0.012**	–0.264	0.784
	Control Correct (#)	0.018	0.238	0.063	0.872	–0.006	0.480	–0.010	0.545	0.459	0.154

*Results from statistical analysis of HDBR + AG assessing effects of HDBR, group, artificial gravity, sex and age. Values that are significant following Benjamini-Hochberg FDR correction are bolded and underlined.*

*DSST, digit symbol substitution test; RFT, rod and frame test; DTC, dual-task cost; RT, reaction time; SWM, spatial working memory; FMT, Functional Mobility Test; SOT-5, Sensory Organization Test 5; SOT-5M, Sensory Organization Test 5 with head movements; EQ Score, Equilibrium score.*

**TABLE 2:** Recovery from HDBR + AG environments.

Recovery		HDBR		Group		HDBR × AG		Age		Sex	
Sensorimotor Task		β	*p*	β	*p*	β	*p*	β	*p*	β	*p*
FMT	Time (s)	–0.871	**0.0013**	–30.014	0.128	0.475	0.114	0.161	0.110	–6.926	0.001
SOT-5M	EQ Score	0.941	**0.0432**	11.354	0.750	–0.177	0.741	–0.323	0.301	8.761	0.148

*Results from statistical analysis of Recovery assessing effects of HDBR, group, artificial gravity, sex and age. Values that are significant following Benjamini-Hochberg FDR correction are bolded and underlined.*

*FMT, Functional Mobility Test; SOT-5M, Sensory Organization Test 5 with head movements; EQ Score, Equilibrium score.*

**TABLE 3:** Effect of Prei-centrifugation.

Per-centrifugation		HDBR		Group		HDBR × AG		Age		Sex	
		β	*p*	β	*p*	β	*p*	β	*p*	β	*p*
PASAT	Time (s)	–0.000	0.283	0.230	0.622	–0.002	**0**.**0001**	–0.002	0.589	–0.211	0.013
PASAT	Correct (#)	0.207	0.0001	3.821	**0.0154**	–0.050	0.010	0.154	0.205	6.164	0.013
Post-AG Motion	Time	0.348	0.860	8.223	**0.036**	–0.114	0.943	–0.141	0.459	6.326	0.428
Motion Sickness Response	Response (#)	–0.017	0.080	–0.792	**0.040**	0.016	**0.032**	–0.020	0.422	0.660	0.179

**Peri-Centrifugation *Post Hoc***		**HDBR**		**Group**		**HDBR + AG**		**Age**		**Sex**	
PASAT	Time (s)	0.001	0.527	0.092	0.400	–0.003	0.0032	–0.002	0.640	–0.280	0.017
PASAT	Correct (#)	0.077	0.1546	–0.1081	0.711	0.0283	0.407	–0.155	0.275	8.774	0.0069
Motion Sickness Response	Response (#)	–0.017	0.336	–2.197	0.002	0.0161	0.155	–0.025	0.284	–0.538	0.244

*Results from statistical analysis of centrifugation assessing effects of HDBR, group, artificial gravity, sex and age. Values that are significant following Benjamini-Hochberg FDR correction are bolded and underlined. Post Hoc analysis were conducted between the AG groups without the control group. Values that are significant following post hoc analysis are bolded.*

*PASAT, Paced Auditory Serial Addition Test.*

##### The Effect of the HDBR + AG Environment

In the pre/late-HDBR model, time was considered as a continuous variable to assess the effect of the AG intervention on performance. Group was entered as a dependent variable, whereas age, sex, and days in HDBR were entered into the model as covariates. For most measures, such as the cognitive measures and Purdue Pegboard, we assessed performance 7 days prior to entering HDBR (BDC-7), 29 days in HDBR (HDBR29) and 58 days in HDBR (HDBR58). The SOT and FMT require the participant to be in upright stance, which is not allowed during the strict HDBR period. Thus, they were assessed pre- and post-HDBR. The FMT was collected on BDC-7, and the day of exiting HDBR (R + 0). The SOT-5 was collected 1 day prior to entering HDBR (BDC-1) and on R + 0.

##### Recovery From the HDBR + AG Environment

This model was only applied in cases where there were significant changes from pre- to late HDBR + AG, in order to assess recovery. Here, time was considered as a continuous variable to assess the recovery profile. Group was entered as a dependent variable, whereas age, sex, and days in HDBR were entered into the model as covariates.

##### Direct Effects of Centrifugation

This model utilized time as a continuous variable to evaluate performance changes during the 60 days of HDBR while the participants are experiencing, or immediately following, AG. Only the peri-centrifugation metrics (PASAT, motion sickness response, post rotary motion illusion duration) were included in this analysis. Group was entered as a dependent variable, whereas age, sex, and days in HDBR were entered into the model as covariates.

## Results

The results of all statistical models are presented in Tables 1–3, where findings that are bolded and underlined were initially significant and remained so following the Benjamini-Hochberg correction.

### The Effect of the HDBR + AG Environment

**FIGURE 2:**
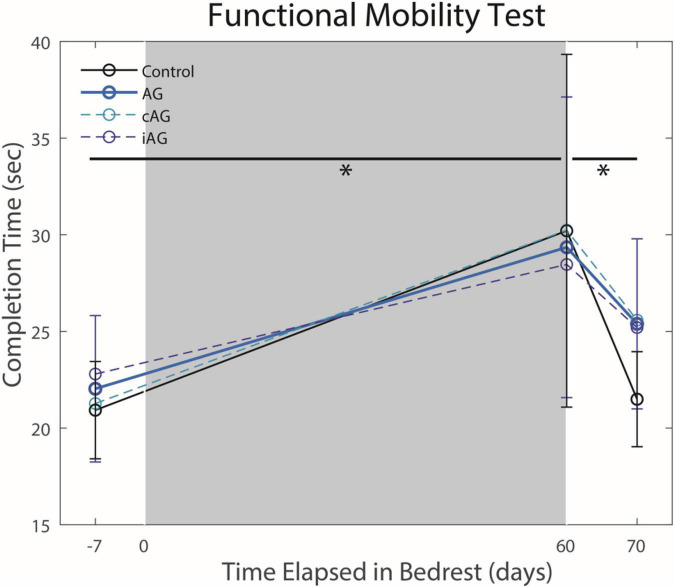
Functional Mobility Test (FMT) performance changes pre- to post-HDBR and post-HDBR recovery. HDBR resulted in a significant increase in completion time (*p* = 0.0002) for all subjects regardless of AG group. Completion time recovered to pre-HDBR levels by 10 days post-HDBR (*p* = 0.013). Significant differences are noted by *.

**FIGURE 3:**
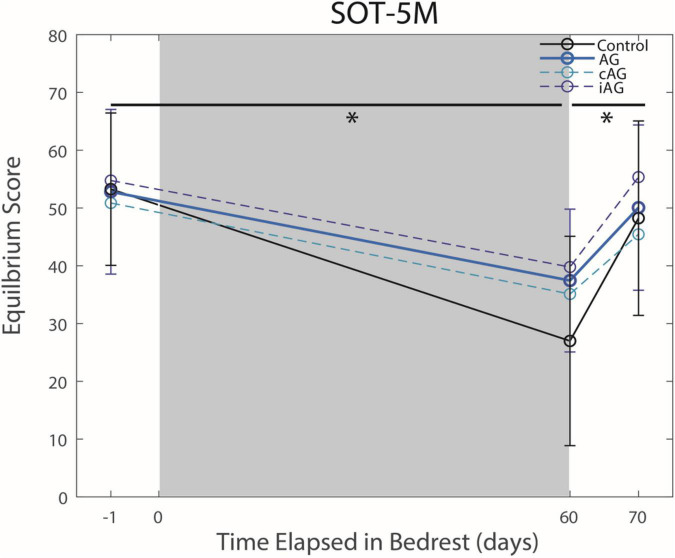
Balance (SOT-5M) changes from pre-HDBR to post-HDBR and post-HDBR recovery. The Sensory Organization Task 5 with head movements (SOT-5M) performance changes in Equilibrium Score indicate an effect of HDBR on balance performance for all groups regardless of AG (*p* = 0.0002). There was a significant recovery of performance post-HDBR (*p* = 0.043). Significant differences are noted by *.

There were no interaction effects between the AG versus control groups on HDBR performance (Table 1). Results indicate a significant main effect from pre to post-HDBR in FMT performance (*p* = 0.0002, Figure 2), reflecting an increase in total completion time following HDBR. There was also a significant main effect for the SOT-5M test, as posture control performance decreased following HDBR (*p* = 0.0002, Figure 3). There was not a significant interaction with AG group; however, the initial post-bedrest equilibrium score was 10.4 points greater for those who underwent AG relative to the controls. There were no significant changes in cognitive measures related to HDBR or AG group.

### Recovery From HDBR + AG

There was a significant main effect seen as an improvement in FMT completion time between the first and second post-bedrest test (Figure 2; *p* = 0.0013). Additionally, there was also a similar significant main effect in SOT-5M equilibrium scores (Figure 3; *p* = 0.043), as participants recovered performance toward pre-HDBR levels.

### Effects of Peri-Centrifugation

**FIGURE 4:**
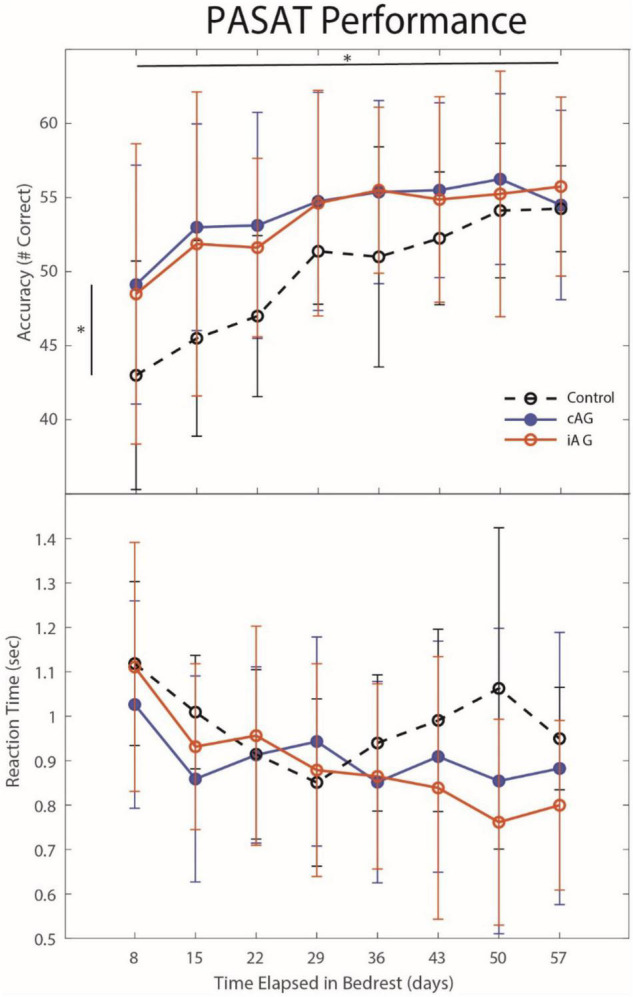
Paced Auditory Serial Addition Task (PASAT) changes during HDBR. PASAT accuracy results indicate that both AG groups performed with higher accuracy throughout HDBR (*p* = 0.015) than the control group, although all groups increased accuracy through HDBR (*p* = 0.0001). There was also a significant group by time interaction (*p* = 0.01) for PASAT reaction times. Significant differences are noted by *.

**FIGURE 5:**
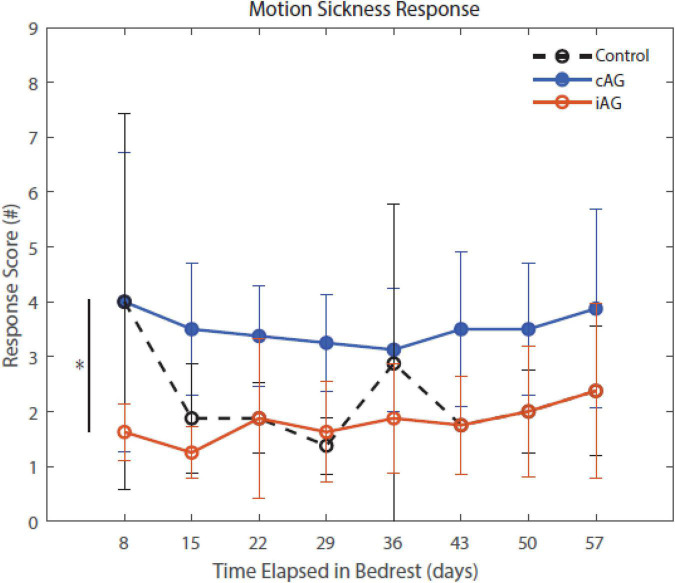
Motion Sickness Response throughout HDBR. Motion sickness response scores indicate a significant group difference between the two AG groups, cAG and iAG, that is maintained throughout the entirety of HDBR (*p* = 0.04). This group difference is noted by the * in the figure. The iAG group had less motion sickness than the cAG group. There is also a significant group by HDBR interaction of these two AG groups (*p* = 0.032).

**FIGURE 6:**
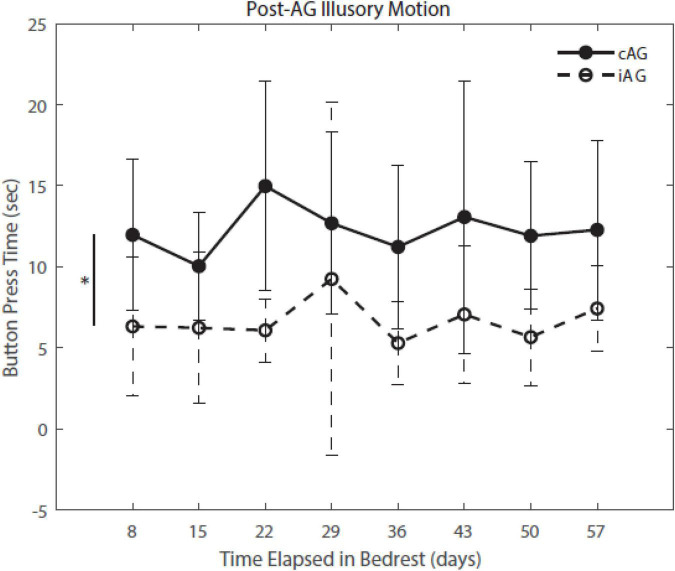
Post-AG Illusory Motion throughout HDBR. Post-AG response times indicate that iAG had a lower level of post-AG illusory motion (*p* = 0.036), indicating that they perceived coming to a stop sooner than the cAG participants.

Results (Table 3) indicate a significant interaction effect of HDBR and AG on both PASAT accuracy (*p* = 0.01) and reaction time (*p* = 0.0001); both iAG and cAG groups were more accurate and responded more quickly than controls, who performed the task outside of the centrifuge (Figure 4). A significant main effect was identified in PASAT accuracy (*p* = 0.0001), with accuracy increasing over the duration of HDBR for all groups. Main effects of sex were also found on both PASAT accuracy (*p* = 0.013) and reaction time (*p* = 0.013). There was a significant HDBR + AG interaction (*p* = 0.032) and main effect of group (*p* = 0.040; Figure 4) identified in motion sickness response. To further examine this, we removed the control group from analysis to determine if the two AG groups were different. The *post hoc* analysis revealed that the continuous AG group having an overall higher self-reported motion sickness score than the intermittent group (*p* = 0.002; Figure 5). A main effect for group in post-AG rotary motion was also identified; the cAG group took significantly longer to perceive that they had come to a full stop following centrifugation (*p* = 0.05; Figure 6).

## Discussion

Here, we investigated the efficacy of artificial gravity applied by short-arm centrifugation to counteract the sensorimotor and cognitive declines associated with HDBR, a standard spaceflight analog environment. As is typical, performance on several tests declined with HDBR. Thirty minutes of daily AG did not mitigate these declines on tasks measured following HDBR. However, participants that received AG performed better on tasks administered during or immediately following centrifugation compared to those who did not receive AG and performed the same tasks. Moreover, those that received AG intermittently in six daily bouts of 5 mins tolerated centrifugation better and experienced less post-AG illusory motion than those that received it continuously.

### Sensorimotor Performance

Previous bed rest studies have identified sensorimotor declines in mobility and balance, as well as declines in fine motor control (Koppelmans et al., 2017; Miller et al., 2018; Lee et al., 2019). Those declines have been shown to model the functional declines of astronauts following long duration spaceflight. Here, we found that FMT and SOT-5M performance decreased as a result of HDBR. Thirty minutes of daily AG did not significantly mitigate these declines, nor did subjects that received AG during HDBR recover to a greater magnitude post bed rest in our measures. While there was no statistically significant effect of 30 mins of daily AG on these balance and mobility measures, it is notable that the AG group’s decline in their SOT-5M equilibrium score with HDBR was 10.4 points less than that of control participants. Moreover, a recent study from the same campaign showed that some postural control measures declined less in bed rest for participants that received AG (De Martino et al., 2021). Thus, a higher dose of AG (in terms of duration and/or magnitude) may prove beneficial. Alternatively, this could be due to the limited sample size, as the effect size for a group comparison on SOT-5M performance between the AG and CTRL post-HDBR is 0.67. This is a large effect size that could be detected in future, larger studies. Additionally, here, the subjects were exposed only to passive AG as they were not actively moving while on the centrifuge. It is possible that if they were required to perform a sensorimotor task during centrifugation that this could alter the results. Exercise in addition to centrifugation may also increase the limited effect of AG, as previous investigations have shown promising results (Wood et al., 2011; Diaz-Artiles, 2015; Diaz-Artiles et al., 2018; Mulavara et al., 2018; English et al., 2019). Overall, AG did not have a negative effect on any of our measures.

### Cognition

Head-down tilt bed rest is frequently used as a spaceflight analog as it replicates the axial body unloading, headward fluid shifts and sensory reweighting that are seen with spaceflight (Roberts et al., 2015; Hargens and Vico, 2016). When assessing cognitive-motor dual-tasking abilities in a prior 70 day bed rest campaign, we identified lower dual-tasking performance in subjects that underwent HDBR, but a greater improvement in counting accuracy while dual tasking in HDBR compared to controls who did not enter HDBR (Yuan et al., 2016). Functional MRI collected during dual-tasking identified that during HDBR the same subjects had higher brain activity in frontal, parietal and cingulate cortices. Others have investigated the effects of 12° HDBR on cognition, finding only small effects on cognitive speed (Basner et al., 2018). When we assessed the same dual-tasking measures in the VaPER campaign (which combined 30 days of bed rest with elevated ambient CO_2_), we found decreases in dual task cost of brain activity in the superior frontal gyrus that returned to baseline after exiting HDBR + CO_2_ (Mahadevan et al., 2021). Further, in the same campaign, our group found performance on the digit symbol substitution test was significantly worse, as were card rotation accuracy and amount completed (Lee et al., 2019). In the same HDBR campaign others identified cognitive deficits, such as a change in speed-accuracy tradeoff (participants became slower and more accurate) and shown that HDBR + CO_2_ is associated with decreased performance speed for various cognitive tests; effects were most prominent for sensorimotor assessments (Basner et al., 2021). In the present investigation, we identified no cognitive declines with HDBR, nor any effects of AG. Failing to identify cognitive deficits associated with HDBR is not uncommon, in our previous investigation of 70 days of HDBR with an exercise countermeasure we found no evidence of cognitive deficits with these same measures. As discussed above, we identified cognitive performance changes in the recent VaPER campaign (Lee et al., 2019), however, this would support the notion that those changes were more likely caused by the increased CO_2_ instead of HDBR. Our finding would be in contrast of other work that showed modest changes in cognition related to HDBR, but not related to CO_2_ or AG; predominantly, those finding identified slowing in sensorimotor speed (Basner et al., 2021). It is possible, however, that brain activity may change without task performance declines, due to compensation or substitution of brain networks relied upon (Rothi and Horner, 1983). We collected functional MRI data in the current sample while participants performed several cognitive and sensorimotor tests; thus, we will be able to investigate this in future analyses.

### Centrifugation

Artificial gravity did not appear to have an effect on sensorimotor and cognitive measures throughout HDBR, but it did affect task performance during or immediately following centrifugation. Both AG groups in the current study were significantly more accurate on the PASAT than controls, who performed the test in bed. Additionally, both cAG and iAG groups had faster response times than controls. It is likely that there were effects of learning across the weekly sessions, as we see performance increase with each session. However, the two groups (AG vs CTRL) still performed significantly different across the HDBR period, suggesting that any effect of learning did not interfere. Similar to what has been previously reported (Clément et al., 2015; Linnarsson et al., 2015), we found that continuous AG induced higher motion sickness than intermittent AG. Interestingly, the control subjects had higher initial motion sickness scores, despite not receiving the AG intervention. This is likely an effect of HDBR, as they had also recorded some dizziness at this time. Overall, their scores decrease as they likely adapt to HDBR. Moreover, continuous AG resulted in a longer period of post centrifugation illusory motion than intermittent AG. Overall, performance on tasks during or immediately following centrifugation is higher than when tasks were performed in HDBR. Performance benefits were similar for continuous and intermittent AG, with those undergoing intermittent AG tolerating it better. These effects are somewhat counter to those described in the introduction (Bles et al., 1997; Arya et al., 2007), which found that 1 h of daily AG resulted in acute cognitive declines. Thus, the daily duration of AG may interact with performance. However, we did not assess performance on the PASAT before the participant entered bedrest, making it possible that there were group differences prior to HDBR.

### Limitations

Primary limitations of this study include a small sample size. While this study included more participants overall than several prior HDBR studies (Cassady et al., 2016; Lee et al., 2019), the sample size is still relatively small per group (*n* = 8). Since several effects trended near standard statistical significance thresholds, a larger study may prove more informative. Additionally, our post-HDBR + AG recovery time point was 10 days following the exit of HDBR + AG. HDBR alone has been shown to result in similar transient sensorimotor deficits, with recovery to near baseline levels 24–48 h following the exit of HDBR (Cassady et al., 2016; Miller et al., 2018). Thus, it is possible that we missed some effects of re-adaptation to the normal upright environment.

## Conclusion

In this study we evaluated whether artificial gravity would mitigate cognitive and sensorimotor declines resulting from HDBR. We identified decreases in sensorimotor performance that showed no interaction with AG, and a lack of overall cognitive findings. However, centrifugation was shown to have a direct, acute effect on performance. Participants that received AG intermittently tolerated it better than those that received it in one continuous bout. In future analyses we will examine brain activity changes and their relation to behavioral performance. While we may not see significant differences in our behavioral assessments, it may be that brain activation patterns are changing to compensate for, or as a result of, AG.

## Data Availability Statement

The raw data supporting the conclusions of this article will be made available by the authors, without undue reservation.

## Ethics Statement

The studies involving human participants were reviewed and approved by University of Florida IRB. The patients/participants provided their written informed consent to participate in this study.

## Author Contributions

GT analyzed the behavioral data, created the figures and tables, and wrote the first draft of the manuscript. HM assisted with processing of the data and preparation of the initial manuscript. YD and NB collected and analyzed the initial data. IK participated in project design and software development. SW, JB, AM, and RS designed the project, secured funding and led the interpretation and discussion of the results. All authors participated in revision of the manuscript and approved the submitted version.

## Conflict of Interest

NB, YD, IK, and AM are employed by KBR. The remaining authors declare that the research was conducted in the absence of any commercial or financial relationships that could be construed as a potential conflict of interest.

## Publisher’s Note

All claims expressed in this article are solely those of the authors and do not necessarily represent those of their affiliated organizations, or those of the publisher, the editors and the reviewers. Any product that may be evaluated in this article, or claim that may be made by its manufacturer, is not guaranteed or endorsed by the publisher.

## Footnotes

1
https://www.nasa.gov/sites/default/files/atoms/files/bed_rest_studies_complete.pdf

## References

[B1] Adams G. R. Caiozzo V. J. Baldwin K. M. (2003). Skeletal muscle unweighting: spaceflight and ground-based models. *J. Appl. Physiol.* 95 2185–2201. 10.1152/japplphysiol.00346.2003 14600160

[B2] Anguera J. A. Reuter-Lorenz P. A. Willingham D. T. Seidler R. D. (2010). Contributions of spatial working memory to visuomotor learning. *J. Cogn. Neurosci.* 22 1917–1930. 10.1162/jocn.2009.21351 19803691

[B3] Arya M. Paloski W. H. Young L. R. (2007). Centrifugation protocol for the NASA Artificial Gravity-Bed Rest Pilot Study. *J. Grav. Physiol.* 14 5–8. 18372684

[B4] Banker L. A. Salazar A. P. Lee J. K. Beltran N. E. Kofman I. S. De Dios Y. E. (2021). The effects of a spaceflight analog with elevated CO2 on sensorimotor adaptation. *J. Neurophys.* 125 426–436. 10.1152/jn.00306.2020 33296611

[B5] Basner M. Nasrini J. Hermosillo E. McGuire S. Dinges D. F. Moore T. M. (2018). Effects of -12^°^ head-down tilt with and without elevated levels of CO2 on cognitive performance: the SPACECOT study. *J. Appl. Physiol.* 124 750–760. 10.1152/japplphysiol.00855.2017 29357516

[B6] Basner M. Stahn A. C. Nasrini J. Dinges D. F. Moore T. M. Gur R. C. (2021). Effects of Head-Down Tilt Bed Rest Plus Elevated CO2 on Cognitive Performance. *J. Appl. Physiol.* 2021:865. 10.1152/japplphysiol.00865.2020 PMC8262780 33630672

[B7] Benjamini Y. Hochberg Y. (1995). Controlling the False Discovery Rate: A Practical and Powerful Approach to Multiple Testing. *J. R. Stat. Soc.* 57 289–300. 10.1111/j.2517-6161.1995.tb02031.x

[B8] Black F. O. Paloski W. H. Doxey-Gasway D. D. Reschke M. F. (1995). Vestibular plasticity following orbital spaceflight: recovery from postflight postural instability. *Acta Oto-Laryngolog. Suppl.* 520(Pt 2), 450–454. 10.3109/00016489509125296 8749187

[B9] Black F. O. Paloski W. H. Reschke M. F. Igarashi M. Guedry F. Anderson D. J. (1999). Disruption of postural readaptation by inertial stimuli following space flight. *J. Vest. Res.* 9 369–378. 10.3233/ves-1999-9506 10544375

[B10] Bles W. de Graaf B. Bos J. E. Groen E. Krol J. R. (1997). A sustained hyper-g load as a tool to simulate space sickness. *J. Grav. Physiol.* 4 1–4. 10.1007/978-3-319-50909-9_24-1 11540661

[B11] Bloomberg J. J. Peters B. T. Cohen H. S. Mulavara A. P. (2015). Enhancing astronaut performance using sensorimotor adaptability training. *Front. Syst. Neurosci.* 9:129. 10.3389/fnsys.2015.00129 26441561 PMC4584940

[B12] Bloomberg J. J. Peters B. T. Smith S. L. Huebner W. P. Reschke M. F. (1997). Locomotor head-trunk coordination strategies following space flight. *J. Vest. Res.* 7 161–177. 10.3233/ves-1997-72-307 9178222

[B13] Buckey J. C. Jr. Lane L. D. Levine B. D. Watenpaugh D. E. Wright S. J. Moore W. E. (1996). Orthostatic intolerance after spaceflight. *J. Appl. Physiol.* 81 7–18. 8828642 10.1152/jappl.1996.81.1.7

[B14] Cassady K. Koppelmans V. Reuter-Lorenz P. De Dios Y. Gadd N. Wood S. (2016). Effects of a spaceflight analog environment on brain connectivity and behavior. *NeuroImage* 141 18–30. 10.1016/j.neuroimage.2016.07.029 27423254

[B15] Clément G. R. Boyle R. D. George K. A. Nelson G. A. Reschke M. F. Williams T. J. (2020). Challenges to the central nervous system during human spaceflight missions to Mars. *J. Neurophysiol.* 123 2037–2063. 10.1152/jn.00476.2019 32292116

[B16] Clément G. R. Bukley A. P. Paloski W. H. (2015). Artificial gravity as a countermeasure for mitigating physiological deconditioning during long-duration space missions. *Front. Syst. Neurosci.* 9:92. 10.3389/fnsys.2015.00092 26136665 PMC4470275

[B17] Clément G. Skinner A. Lathan C. (2013). Distance and Size Perception in Astronauts during Long-Duration Spaceflight. *Life* 3 524–537. 10.3390/life3040524 25369884 PMC4187133

[B18] De Martino E. Salomoni S. E. Hodges P. W. Hides J. Lindsay K. Debuse D. (2021). Intermittent short-arm centrifugation is a partially effective countermeasure against upright balance deterioration following 60-day head-down tilt bed rest. *J. Appl. Physiol.* 2021:180. 10.1152/japplphysiol.00180.2021 34197228

[B19] Diaz-Artiles A. (2015). *Exercise Under Artificial Gravity-Experimental and Computational Approaches.* Ph.D. thesis. Cambridge, MA: Massachusetts Institute of Technology.

[B20] Diaz-Artiles A. Heldt T. Young L. R. (2018). Short-term cardiovascular response to short-radius centrifugation with and without ergometer exercise. *Front. Physiol.* 9:1492. 10.3389/fphys.2018.01492 30483141 PMC6242912

[B21] DiZio P. Lackner J. R. (2002). Sensorimotor aspects of high-speed artificial gravity: III. Sensorimotor adaptation. *J. Vest. Res.* 12 291–299. 10.3233/ves-2003-125-609 14501105

[B22] Ekstrom R. B. Harman H. H. (1976). *Manual for kit of factor-referenced cognitive tests, 1976.* Princeton, NJ: Educational testing service.

[B23] English K. L. Bloomberg J. J. Mulavara A. P. Ploutz-Snyder L. L. (2019). Exercise Countermeasures to Neuromuscular Deconditioning in Spaceflight. *Compr. Physiol.* 10 171–196. 10.1002/cphy.c190005 31853963

[B24] Frett T. Green D. A. Mulder E. Noppe A. Arz M. Pustowalow W. (2020). Tolerability of daily intermittent or continuous short-arm centrifugation during 60-day 6o head down bed rest (AGBRESA study). *PLoS One* 15:e0239228. 10.1371/journal.pone.0239228 32946482 PMC7500599

[B25] Garrett-Bakelman F. E. Darshi M. Green S. J. Gur R. C. Lin L. Macias B. R. (2019). The NASA Twins Study: A multidimensional analysis of a year-long human spaceflight. *Science* 364:6436. 10.1126/science.aau8650 PMC7580864 30975860

[B26] Gianaros P. J. Muth E. R. Mordkoff J. T. Levine M. E. Stern R. M. (2001). A questionnaire for the assessment of the multiple dimensions of motion sickness. *Aviat. Space Env. Med.* 72 115–119. 11211039 PMC2910410

[B27] Gronwall D. M. (1977). Paced auditory serial-addition task: a measure of recovery from concussion. *Percept. Mot. Skills* 44 367–373. 10.2466/pms.1977.44.2.367 866038

[B28] Hargens A. R. Vico L. (2016). Long-duration bed rest as an analog to microgravity. *J. Appl. Physiol.* 120 891–903. 10.1152/japplphysiol.00935.2015 26893033

[B29] Hupfeld K. E. Lee J. K. Gadd N. E. Kofman I. S. De Dios Y. E. Bloomberg J. J. (2019). Neural Correlates of Vestibular Processing During a Spaceflight Analog With Elevated Carbon Dioxide (CO2): A Pilot Study. *Front. Syst. Neurosci.* 13:80. 10.3389/fnsys.2019.00080 31998084 PMC6965349

[B30] Hupfeld K. E. McGregor H. R. Lee J. K. Beltran N. E. Kofman I. S. De Dios Y. E. (2020). The Impact of 6 and 12 Months in Space on Human Brain Structure and Intracranial Fluid Shifts. *Cereb. Cort. Comm.* 1:tgaa023. 10.1093/texcom/tgaa023 32864615 PMC7446230

[B31] Kanas N. Manzey D. (2008). “Basic Issues of Human Adaptation to Space Flight,” in *Space Psychology and Psychiatry*, eds Kanas N. Manzey D. (Netherlands: Springer), 15–48. 10.1007/978-1-4020-6770-9_2

[B32] Koppelmans V. Bloomberg J. J. De Dios Y. E. Wood S. J. Reuter-Lorenz P. A. Kofman I. S. (2017). Brain plasticity and sensorimotor deterioration as a function of 70 days head down tilt bed rest. *PLo*S *One* 12:e0182236. 10.1371/journal.pone.0182236 28767698 PMC5540603

[B33] Koppelmans V. Erdeniz B. De Dios Y. E. Wood S. J. Reuter-Lorenz P. A. Kofman I. (2013). Study protocol to examine the effects of spaceflight and a spaceflight analog on neurocognitive performance: extent, longevity, and neural bases. *BMC Neurol.* 13:205. 10.1186/1471-2377-13-205 24350728 PMC3878338

[B34] Kornilova L. N. (1997). Vestibular function and sensory interaction in altered gravity. *Adv. Space Biol. Med.* 6 275–313. 10.1016/s1569-2574(08)60087-8 9048143

[B35] Lackner J. R. DiZio P. (1996). Motor function in microgravity: movement in weightlessness. *Curr. Opin. Neurob.* 6 744–750. 10.1016/s0959-4388(96)80023-7 9000028

[B36] Lackner J. R. DiZio P. (1998). Adaptation in a rotating artificial gravity environment. *Brain Res. Rev.* 28, 194–202. 9795214 10.1016/s0165-0173(98)00039-3

[B37] Layne C. S. Lange G. W. Pruett C. J. McDonald P. V. Merkle L. A. Mulavara A. P. (1998). Adaptation of neuromuscular activation patterns during treadmill walking after long-duration space flight. *Acta Astronaut.* 43 107–119. 10.1016/s0094-5765(98)00148-9 11541918

[B38] Lee J. K. De Dios Y. Kofman I. Mulavara A. P. Bloomberg J. J. Seidler R. D. (2019). Head Down Tilt Bed Rest Plus Elevated CO2 as a Spaceflight Analog: effects on Cognitive and Sensorimotor Performance. *Front. Hum. Neurosci.* 13:355. 10.3389/fnhum.2019.00355 31680909 PMC6811492

[B39] Linnarsson D. Hughson R. L. Fraser K. S. Clément G. Karlsson L. L. Mulder E. (2015). Effects of an artificial gravity countermeasure on orthostatic tolerance, blood volumes and aerobic power after short-term bed rest (BR-AG1). *J. Appl. Physiol.* 118 29–35. 10.1152/japplphysiol.00061.2014 25342708

[B40] Lowrey C. R. Perry S. D. Strzalkowski N. D. J. Williams D. R. Wood S. J. Bent L. R. (2014). Selective skin sensitivity changes and sensory reweighting following short-duration space flight. *J. Appl. Physiol.* 116 683–692. 10.1152/japplphysiol.01200.2013 24458748

[B41] Mahadevan A. D. Hupfeld K. E. Lee J. K. De Dios Y. E. Kofman I. S. Beltran N. E. (2021). Head-Down-Tilt Bed Rest With Elevated CO2: Effects of a Pilot Spaceflight Analog on Neural Function and Performance During a Cognitive-Motor Dual Task. *Front. Physiol.* 12:654906. 10.3389/fphys.2021.654906 34512371 PMC8424013

[B42] Manzey D. Lorenz B. (1998). Mental performance during short-term and long-term spaceflight. *Brain Res.* 28 215–221.s 10.1016/s0165-0173(98)00041-1 9795225

[B43] Manzey D. Lorenz B. Schiewe A. Finell G. Thiele G. (1995). Dual-task performance in space: results from a single-case study during a short-term space mission. *Hum. Fact.* 37 667–681. 10.1518/001872095778995599 8851772

[B44] McDonald P. V. Basdogan C. Bloomberg J. J. Layne C. S. (1996). Lower limb kinematics during treadmill walking after space flight: implications for gaze stabilization. *Exp. Brain Res.* 112 325–334. 10.1007/BF00227650 8951400

[B45] McGregor H. R. Lee J. K. Mulder E. De Dios Y. Beltran N. E. (2020). Ophthalmic Changes in a Spaceflight Analog Are Associated with Brain Functional Reorganization. *bioRxiv* 2020:289827. 10.1101/2020.09.09.289827v1.abstract PMC8357001 34105833

[B46] McGregor H. R. Lee J. K. Mulder E. R. De Dios Y. E. Beltran N. E. Kofman I. S. (2021). Brain connectivity and behavioral changes in a spaceflight analog environment with elevated CO2. *NeuroImage* 225:117450. 10.1016/j.neuroimage.2020.117450 33075558

[B47] Miller C. A. Kofman I. S. Brady R. R. May-Phillips T. R. Batson C. D. Lawrence E. L. (2018). Functional Task and Balance Performance in Bed Rest Subjects and Astronauts. *Aerospace Med. Hum. Perf.* 89 805–815. 10.3357/AMHP.5039.2018 30126513

[B48] Moore S. T. MacDougall H. G. Paloski W. H. (2010). Effects of head-down bed rest and artificial gravity on spatial orientation. *Exp. Brain Res.* 204 617–622.s 10.1007/s00221-010-2317-0 20535455

[B49] Mulavara A. P. Feiveson A. H. Fiedler J. Cohen H. Peters B. T. Miller C. (2010). Locomotor function after long-duration space flight: effects and motor learning during recovery. *Exp. Brain Res.* 202 649–659. 20135100 10.1007/s00221-010-2171-0

[B50] Mulavara A. P. Peters B. T. Miller C. A. Kofman I. S. Reschke M. F. Taylor L. C. (2018). Physiological and Functional Alterations after Spaceflight and Bed Rest. *Med. Sci. Sports Exerc.* 50 1961–1980. 10.1249/MSS.0000000000001615 29620686 PMC6133205

[B51] Mulder E. Linnarsson D. Paloski W. H. Rittweger J. Wuyts F. L. Zange J. (2014). Effects of five days of bed rest with and without exercise countermeasure on postural stability and gait. *J. Muscul. Neur. Inter.* 14 359–366. 25198232

[B52] Nashner L. M. (1972). Vestibular postural control model. *Kybernetik* 10 106–110. 10.1007/BF00292236 4537349

[B53] Newman D. J. Jackson D. K. Bloomberg J. J. (1997). Altered astronaut lower limb and mass center kinematics in downward jumping following space flight. *Exp. Brain Res.* 117 30–42. 10.1007/pl00005788 9386002

[B54] Paloski W. H. Bloomberg J. J. Reschke M. F. Harm D. L. (1994). Spaceflight-induced changes in posture and locomotion. *J. Biomech.* 27:812. 10.1016/0021-9290(94)91366-8s

[B55] Paloski W. H. Reschke M. F. Black F. O. Doxey D. D. Harm D. L. (1992). Recovery of postural equilibrium control following spaceflight. *Ann. N Y Acad. Sci.* 656 747–754. 10.1111/j.1749-6632.1992.tb25253.x 1599180

[B56] Pinheiro J. Bates D. DebRoy S. Sarkar D. R Core Team (2022). *nlme: Linear and Nonlinear Mixed Effects Models. R Package Version 3.1-155.* Available online at: https://CRAN.R-project.org/package=nlme (accessed January 13, 2022).

[B57] Ploutz-Snyder L. L. Downs M. Goetchius E. Crowell B. English K. L. Ploutz-Snyder R. (2018). Exercise Training Mitigates Multisystem Deconditioning during Bed Rest. *Medicine and Science in Sports and Exercise* 50 1920–1928. 10.1249/MSS.0000000000001618 29924746 PMC6647016

[B58] R Core Team (2019). *R: A Language and Environment for Statistical Computing.* Vienna: R Foundation for Statistical Computing.

[B59] Reschke M. F. Bloomberg J. J. Harm D. L. Paloski W. H. (1994a). Space flight and neurovestibular adaptation. *J. Clin. Pharm.* 34 609–617. 10.1002/j.1552-4604.1994.tb02014.x 8083392

[B60] Reschke M. F. Bloomberg J. J. Paloski W. H. Harm D. L. Parker D. E. (1994b). *Neurophysiologic aspects: sensory and sensorimotor function in space physiology and medicine*, eds Nicogossian A. E. Huntoon C. L. Pool S. L. (Philadelphia: Lea & Febiger).

[B61] Reschke M. F. Bloomberg J. J. Harm D. L. Paloski W. H. Layne C. McDonald V. (1998). Posture, locomotion, spatial orientation, and motion sickness as a function of space flight. *Brain Res.* 28 102–117. 10.1016/s0165-0173(98)00031-9 9795167

[B62] Reschke M. F. Bloomberg J. J. Paloski W. H. Mulavara A. P. Feiveson A. H. Harm D. L. (2009). Postural reflexes, balance control, and functional mobility with long-duration head-down bed rest. *Aviat. Space Env. Med.* 5(Suppl.), A45–A54. 10.3357/asem.br06.2009 19476169

[B63] Roberts D. R. Albrecht M. H. Collins H. R. Asemani D. Chatterjee A. R. Spampinato M. V. (2017). Effects of Spaceflight on Astronaut Brain Structure as Indicated on MRI. *New Engl. J. Med.* 377 1746–1753. 10.1056/NEJMoa1705129 29091569

[B64] Roberts D. R. Zhu X. Tabesh A. Duffy E. W. Ramsey D. A. Brown T. R. (2015). Structural brain changes following long-term 6 head-down tilt bed rest as an analog for spaceflight. *AJNR* 36 2048–2054. 10.3174/ajnr.A4406 26185326 PMC7964872

[B65] Rothi L. J. Horner J. (1983). Restitution and substitution: two theories of recovery with application to neurobehavioral treatment. *J. Clin. Neuropsychol.* 5 73–81. 10.1080/01688638308401152 6826766

[B66] Salazar A. P. Hupfeld K. E. Lee J. K. Banker L. A. Tays G. D. Beltran N. E. (2021). Visuomotor Adaptation Brain Changes During a Spaceflight Analog With Elevated Carbon Dioxide (CO2): a Pilot Study. *Front. Neural Circ.* 15:659557. 10.3389/fncir.2021.659557 34163332 PMC8215599

[B67] Salazar A. P. Hupfeld K. E. Lee J. K. Beltran N. E. Kofman I. S. De Dios Y. E. (2020). Neural Working Memory Changes During a Spaceflight Analog With Elevated Carbon Dioxide: a Pilot Study. *Front. Syst. Neurosci.* 14:48. 10.3389/fnsys.2020.00048 32848641 PMC7399639

[B68] Seaton K. A. Slack K. J. Sipes W. Bowie K. (2007). Artificial gravity as a multi-system countermeasure: effects on cognitive function. *J. Grav. Physiol.* 14 27–30. 18372688

[B69] Shepard S. Metzler D. (1988). Mental rotation: effects of dimensionality of objects and type of task. *J. Exp. Psychol.* 14 3–11.s 2964504

[B70] Stavnichuk M. Mikolajewicz N. Corlett T. Morris M. Komarova S. V. (2020). A systematic review and meta-analysis of bone loss in space travelers. *NPJ Microgr.* 6:13. 10.1038/s41526-020-0103-2 32411816 PMC7200725

[B71] Tiffin J. Asher E. J. (1948). The Purdue pegboard; norms and studies of reliability and validity. *J. Appl. Psychol.* 32 234–247. 10.1037/h0061266 18867059

[B72] Tombu M. Jolicoeur P. (2003). A central capacity sharing model of dual-task performance. *J. Exp. Psychol.* 29 3–18. 10.1037//0096-1523.29.1.3 12669744

[B73] Welch R. B. Hoover M. Southward E. F. (2009). Cognitive performance during prismatic displacement as a partial analogue of “space fog.”. *Aviat. Space Env. Med.* 80 771–780. 10.3357/asem.2415.2009 19750873

[B74] Weschler D. (1986). *Wechsler Adult Intelligence Scale–Revised UK Edition.* San Antonio, TX: The Psychological Corporation.

[B75] Witkin H. A. Asch S. E. (1948). Studies in space orientation; perception of the upright in the absence of a visual field. *J. Exp. Psychol.* 38 603–614. 10.1037/h0055372 18885709

[B76] Wood S. J. Loehr J. A. Guilliams M. E. (2011). Sensorimotor reconditioning during and after spaceflight. *NeuroRehabilitation* 29 185–195. 10.3233/NRE-2011-0694 22027081

[B77] Wood S. J. Paloski W. H. Clark J. B. (2015). Assessing Sensorimotor Function Following ISS with Computerized Dynamic Posturography. *Aerosp. Med. Hum. Perf.* 12(Suppl.), A45–A53. 10.3357/AMHP.EC07.2015 26630195

[B78] Wood S. J. Reschke M. F. Owen Black F. (2012). Continuous equilibrium scores: factoring in the time before a fall. *Gait Posture* 36 487–489. 10.1016/j.gaitpost.2012.04.014 22640866

[B79] Yuan P. Koppelmans V. Reuter-Lorenz P. A. De Dios Y. E. Gadd N. E. Wood S. J. (2016). Increased Brain Activation for Dual Tasking with 70-Days Head-Down Bed Rest. *Front. Syst. Neurosci.* 10:71. 10.3389/fnsys.2016.00071 27601982 PMC4993791

[B80] Yuan P. Koppelmans V. Reuter-Lorenz P. De Dios Y. (2017). *Brain activations for vestibular stimulation and dual tasking change with spaceflight.* Available online at: https://ntrs.nasa.gov/search.jsp?R=20170003871 (accessed date May 2, 2017).

[B81] Yuan P. Koppelmans V. Reuter-Lorenz P. De Dios Y. Gadd N. Riascos R. (2018a). Change of cortical foot activation following 70 days of head-down bed rest. *J. Neurophys.* 119 2145–2152. 10.1152/jn.00693.2017 29488843 PMC6032127

[B82] Yuan P. Koppelmans V. Reuter-Lorenz P. De Dios Y. Gadd N. Wood S. (2018b). Vestibular brain changes within 70 days of head down bed rest. *Hum. Brain Mapp.* 39 2753–2763. 10.1002/hbm.24037 29528169 PMC6033666

